# Supplementary figures and images for: Resistance to ectromelia virus infection requires cGAS in bone marrow-derived cells which can be bypassed with cGAMP therapy
Source: PLoS Pathog. 2019 Dec 26;15(12):e1008239. doi: 10.1371/journal.ppat.1008239 (PMC6974301; doi:10.1371/journal.ppat.1008239)

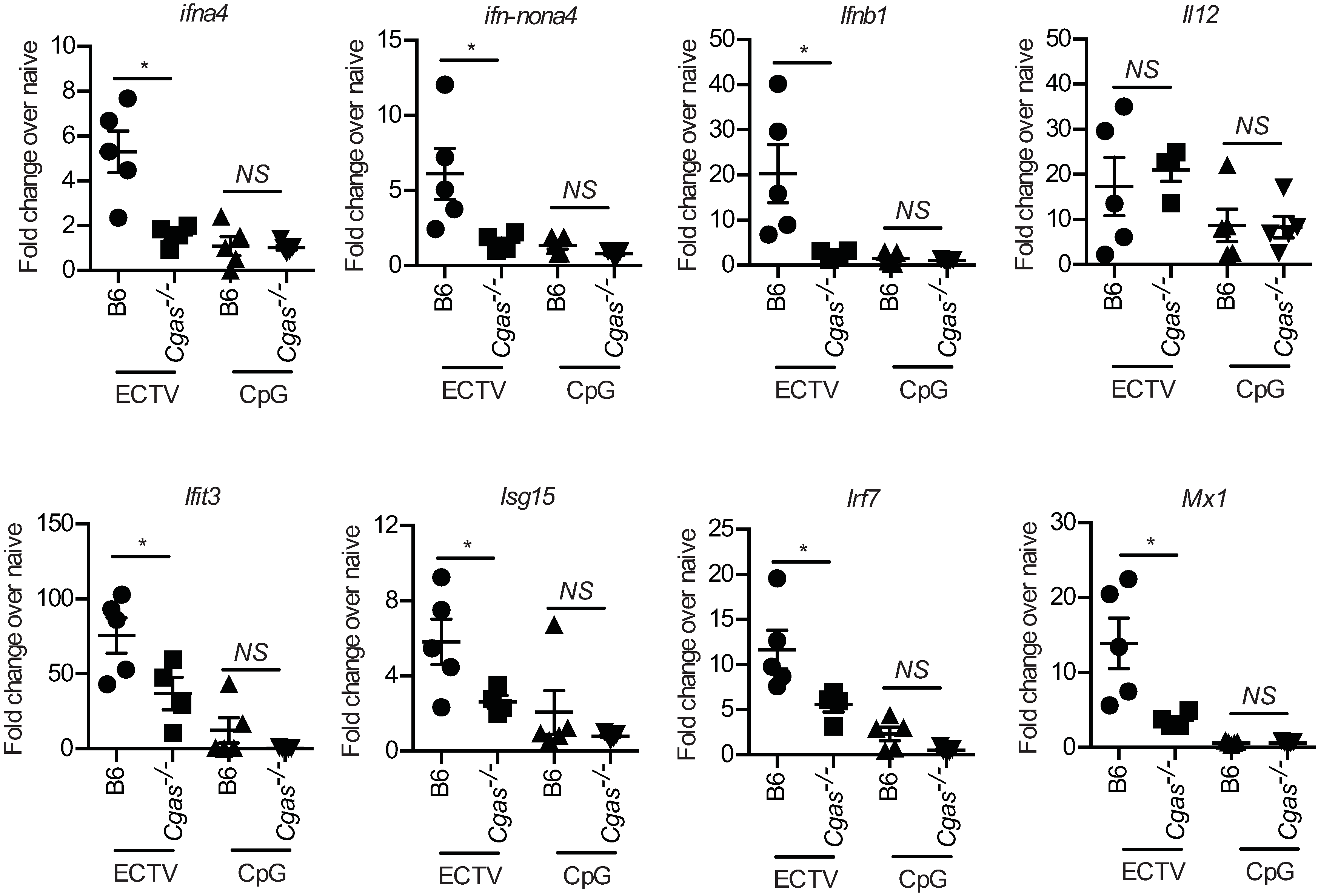

Supplement: S1 Fig — B6 or Cgas-/- mice were either infected with 3,000 pfu ECTV or given 25 ug CpG in the footpad. LNs were harvested at 2 dpi. Data are displayed as mean ± SEM from 5 mice per group in experiment, which is representative of two similar experiments. For all, *p<0.05. (TIF) [file ppat.1008239.s001.tif]

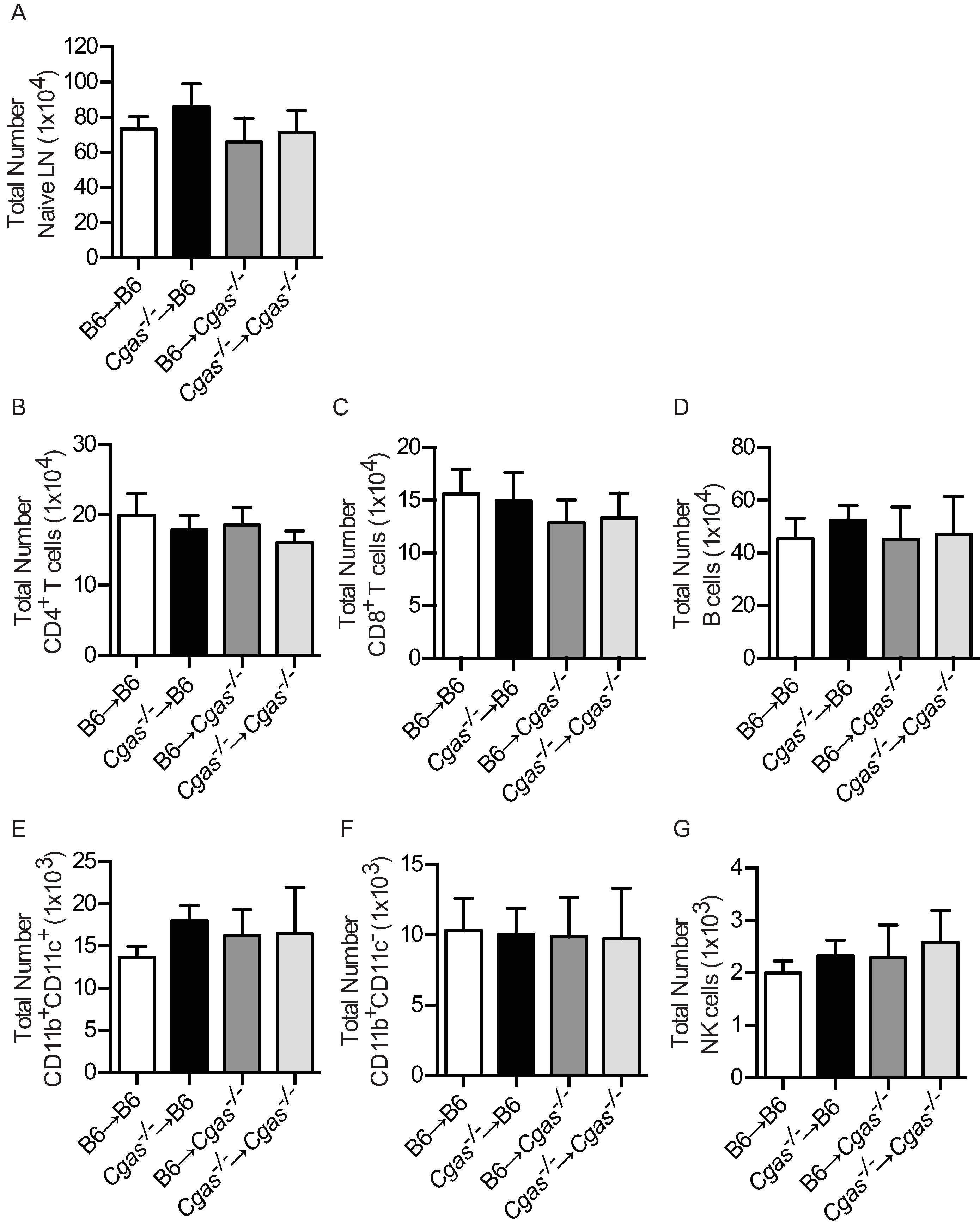

Supplement: S2 Fig — (A) Total cellularity of the naïve contralateral LNs of B6-Cgas-/- chimeric mice. (B-G) Total numbers of the indicated immune cell subtype in the naïve contralateral LNs of B6-Cgas-/- chimeric mice. Data are displayed as mean ± SEM with 10–15 mice per group combined from three similar, independent experiments. (TIF) [file ppat.1008239.s002.tif]

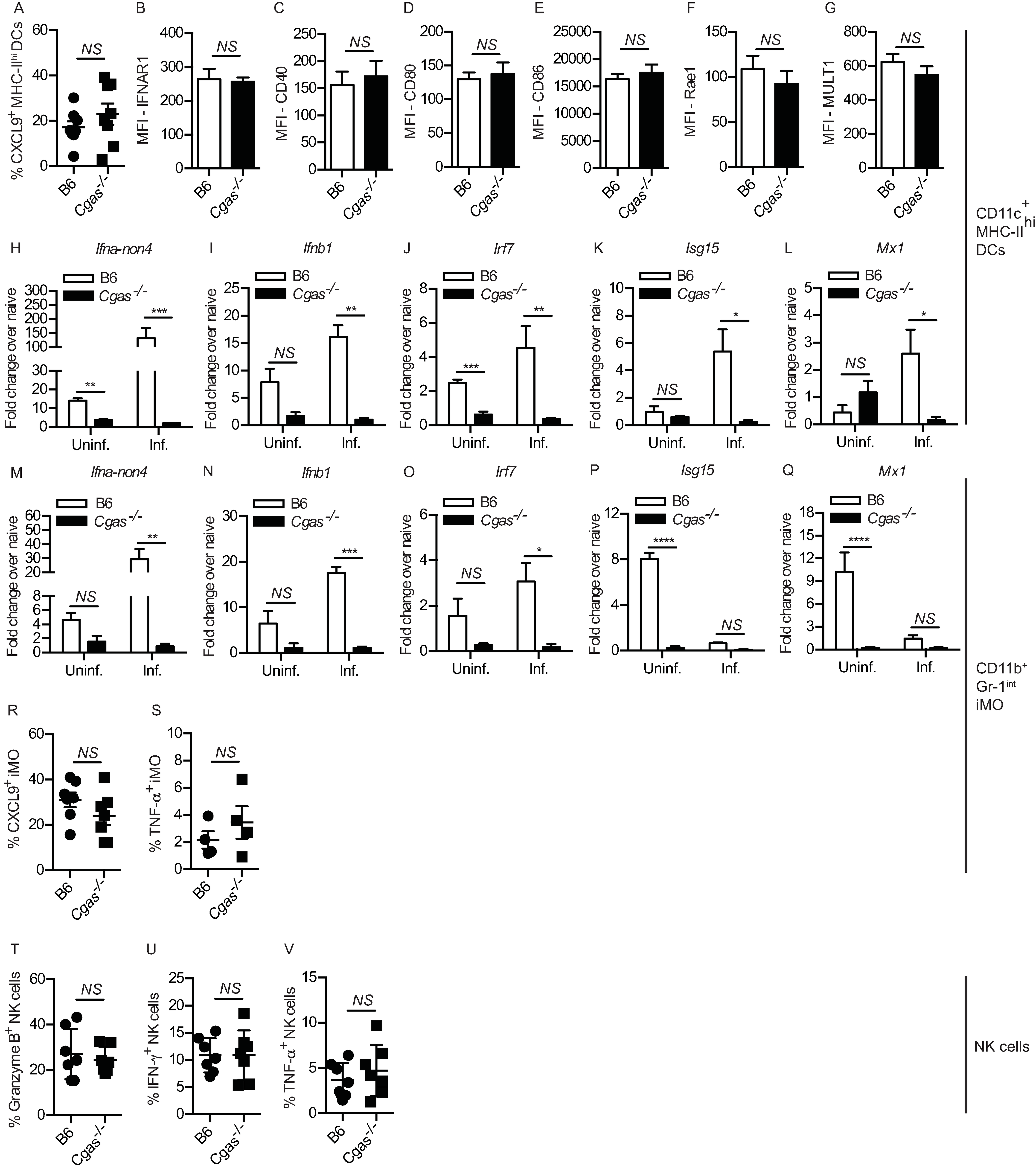

Supplement: S3 Fig — (A) Frequency of CXCL9+MHC-IIhi DCs in the dLN of the indicated mice at 2.5 dpi. (B-G) MFI of IFNAR1 (B), costimulatory molecules CD40 (C), CD80 (D) and CD86 (E), and NKG2D ligands Rae1 (G) and MULT1 (G). Data are displayed as mean ± SEM of 10–12 mice per group combined from three independent experiments. (H-L) Expression of mRNA for Ifna-non4 (H), Ifnb1 (I), Irf7 (J), Isg15 (K) and Mx1 (L) from sorted uninfected and infected MHC-IIhi DCs from the dLN of the indicated mice at 2 dpi. Data are displayed as mean ± SEM of pooled cells from 6–8 mice per group in one experiment, which is representative of two similar experiments. P values were calculated based on three technical replicates. (M-Q) As in (H-L) but for iMO. (R-S) Frequency of CXCL9+ and TNF-α+ iMO in the dLN at 2.5 dpi during ECTV infection. (T-V) Frequencies of Granzyme B+ (T), IFN-γ+ (U), and TNF-α+ (V) NK cells in the dLN of the indicated mice at 2.5 dpi. For all, *p<0.05, **p<0.01, ***p<0.001, ****p<0.0001. (TIF) [file ppat.1008239.s003.tif]

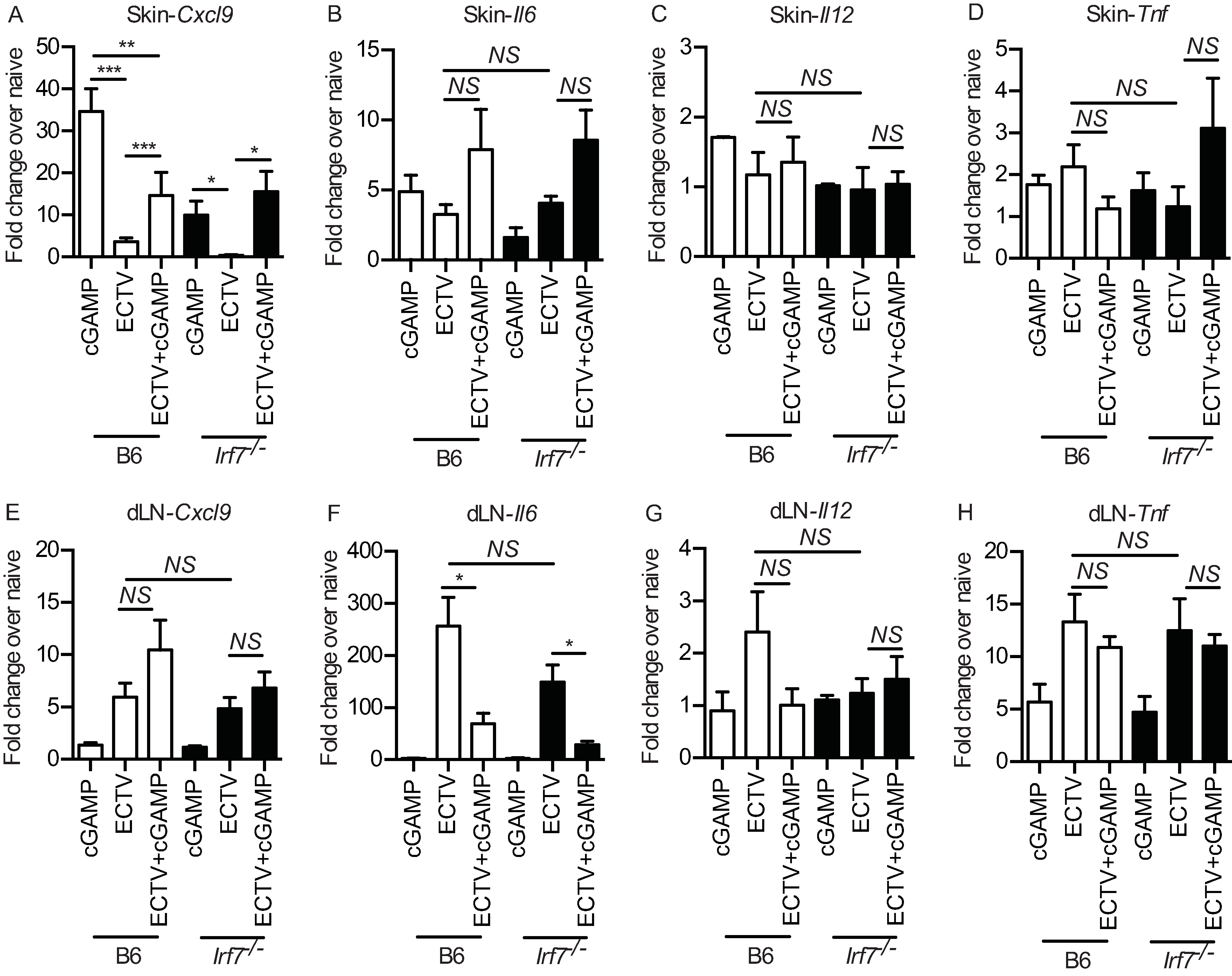

Supplement: S4 Fig — (A-H) Expression of proinflammatory cytokines and chemokines in the skin (A-D) and dLN (E-H) of B6 and Irf7-/- mice at 2 dpi with or without cGAMP administration. Data are displayed as mean ± SEM from 5 mice per group in one experiment, which is representative of three independent experiments. For all, *p<0.05, **p<0.01, ***p<0.001, ****p<0.0001. (TIF) [file ppat.1008239.s004.tif]

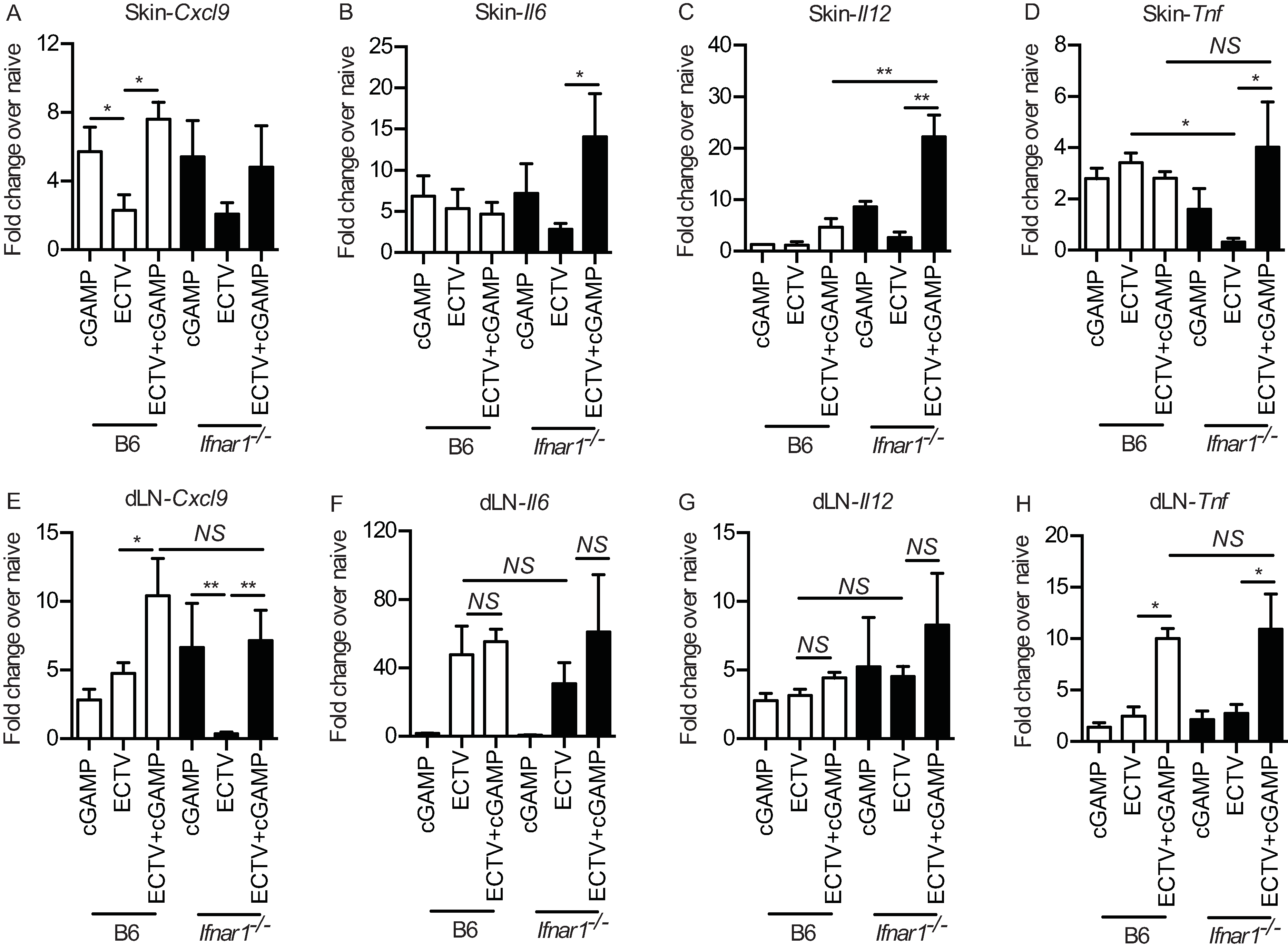

Supplement: S5 Fig — (A-H) Expression of proinflammatory cytokines and chemokines in the skin (A-D) and dLN (E-H) of B6 and Ifnar-/- mice at 2 dpi with or without cGAMP administration. Data are displayed as mean ± SEM from 5 mice per group in one experiment, which is representative of three independent experiments. For all, *p<0.05, **p<0.01, ***p<0.001, ****p<0.0001. (TIF) [file ppat.1008239.s005.tif]
