# Supplementary material for: Resistance to ectromelia virus infection requires cGAS in bone marrow-derived cells which can be bypassed with cGAMP therapy
Source: PLoS Pathog. 2019 Dec 26;15(12):e1008239. doi: 10.1371/journal.ppat.1008239 (PMC6974301; doi:10.1371/journal.ppat.1008239)
Supplement: Attachment — Submitted filename: cGAS PP rebuttal 10-23-19 Final.docx [file ppat.1008239.s006.docx]

10-23-19

# Point by point reply to the reviewers’ comments

We would like to thank the reviewers for their insightful questions and comments regarding our manuscript, “Resistance to an acute viral disease requires cGAS in bone marrow-derived cells which can be bypassed with cGAMP therapy”. The reviewers were positive regarding the overall composition and content of the manuscript. We have carefully followed the reviewers’ helpful suggestions and provided in-depth answers to their questions. This has resulted in improved figures, additional supplementary figures and revised text. Overall, we feel that our revised manuscript is highly responsive to the reviewers’ suggestions and more importantly, represents a significantly improved study that elaborately highlights the mechanistic role of cGAS in bone marrow-derived cells in mediating protective resistance against Ectromelia virus infection and moreover, dissects the integral transcription factors and receptors necessary for cGAMP-mediated protective therapy. Please see below for a point-by-point reply to the reviewers’ comments.

## Part II – Major Issues: Key Experiments Required for Acceptance

**Reviewer #1:**

Figure 2: Is the difference in IFN-I and ISG dependent on the challenge? What if you challenged with TLR9 agonist CpG? Does the difference persist? This is an important control to test if it is truly an effect of CpG being engaged by the virus – or – a basal lack of IFN-I/ISGs due Cgas deficiency.

Reviewer 1 brings up an excellent point regarding whether the decrease in IFN-I/ISG expression is dependent on the specific type of challenge/infection model. We followed Reviewer 1’s suggestion of using CpG in *Cgas^-/-^* mice. As shown below in **Reviewer Figure 1**, injection of 25 ug of CpG into the footpad B6 or *Cgas^-/-^* mice does not induce IFN-I or ISG expression at 2 dpi in either B6 or *Cgas^-/-^* mice (as a positive control for CpG, we measured IL-12 mRNA levels, which was upregulated in both B6 and *Cgas^-/-^* mice). We have also previously found that CpG injection into the footpad induces a large influx of MHC-II^hi^ DCs from the skin to the dLN and concomitant iMO accumulation to the dLN (unpublished data). Furthermore, from what we know from our previous studies and unpublished data, CpG-TLR9 is more important for recruitment of MHC-II^hi^ DCs and iMO to the dLN and appears to be dispensable for IFN-I production, which is dependent on cGAS-STING-IRF7 pathway (Xu et al. 2015 and unpublished data). Finally, we have also previously shown that the majority of IFN-I expression in the dLN is dependent on infected iMO so in lieu of infection (with CpG), it is not entirely surprising that there is a relative lack of IFN-I induction. Overall, these data indicate that the induction of IFN-I/ISGs is dependent on cGAS.

one
***Reviewer Figure 1: CpG does not induce IFN-I and ISGs in the dLN of B6 or Cgas^-/-^ mice.*** *B6 or Cgas^-/-^ mice were either infected with 3,000 pfu ECTV or given 25 ug CpG in the footpad. LNs were harvested at 2 dpi. Data are displayed as mean ± SEM from 5 mice per group in experiment, which is representative of two similar experiments. For all, *p<0.05.*

Figure 3 is the most important figure in distinguishing this work from the work of Cheng et al. However, some important controls and important analysis are missing:

Fig 3A: How successful was the chimera–generation? Have the authors tested if the adoptively transferred bone marrow cells led to a functional hematopoietic compartment? Was there a difference in how well the Cgas KO bone-marrow established itself in the recipient compared with the wt bone-marrow? Do the transferred cells have equal amount of TLR9? / or respond equally well to CpG DNA? What about at the time of infection? Has something changed?

As shown in **Reviewer Figure 2**, the BM chimera generation was successful in terms of donor reconstitution in the irradiated hosts as well as approximately equivalent cellularity and total numbers of different immune cells subsets in the naïve contralateral LNs. We did not perform any experiments in which we measured TLR9 levels or their response to CpG. However, the CpG experiments in **Reviewer Figure 1** indicate that there wouldn’t be a significant IFN-I/ISG response to CpG in the dLN.

***Reviewer Figure 2: Efficiency of B6-Cgas^-/-^ BM chimera reconstitution.*** *(****A****) Total cellularity of the naïve contralateral LNs of B6-Cgas^-/-^ chimeric mice. (****B-G****) Total numbers of the indicated immune cell subtype in the naïve contralateral LNs of B6-Cgas^-/-^ chimeric mice. Data are displayed as mean ± SEM with 10-15 mice per group combined from three similar, independent experiments.*

Figure 4
Fig 4C, L, and T: How can there be “no difference” in percentage of MHC-II^HI^ DCs but a clear difference in total number of cells? This must mean that other cell types must also have increased in numbers. How was the number of cells calculated? Was it per weight of the dLN? Or were the dLNs just different in size between mouse strains? May I missed it in the text, but I can´t find a description of this. This should be clarified. Also, the authors should use dots instead of bars to make clear the distribution of data points.

Reviewer #1 brings up an excellent point regarding the discrepancy between similar percentage but overall decreased total numbers of MHC-II^hi^ DCs (**Figure 4C**), iMOs (**Figure 4L**), and NK cells (**Figure** **4T**). This is due to a significant decrease in size and consequently lower total cell numbers, determined by manual counting with a hemocytometer, in *Cgas^-/-^* dLNs at 2 and 3 dpi, and not an inherent smaller LN size (**Reviewer Figure 3**). This is confirmed with equivalent frequency and total numbers of MHC-II^hi^ DCs, iMOs and NK cells in the dLN of *Cgas^-/-^* mice compared to B6 mice at 1 dpi (**Figure 4C**, **L**, and **T**). We postulated that the decrease in cell numbers in the dLNs at later time points may be attributed to a higher infection rate (**Figure 4F**, **O** and **U**), resulting in an overall increase in cell death in the dLN (**Figure 4V**). We have added additional commentary in the Discussion to provide more clarity.

***Reviewer Figure 3: Total cellularity of ndLN and dLN.*** *Data are displayed as mean ± SEM with 17-22 mice per group combined from five similar, independent experiments. For all, ***p<0.001.*

Reviewer #2:

I do not have any major concerns regarding the technical aspect of the experiments. Most experiments are very well designed, and the results are convincing (although expected).

However, the conceptual and mechanistic novelty of this study are very limited. Almost all of the conclusions are well expected from existing literature on poxvirus sensing by the cGAS-STING pathway, cGAMP is known to stimulates interferon and antiviral response through IRFs and IFNAR. The Cheng et al study (ref 18) last year used the exact same virus ECTV and has already demonstrated many of the similar observations. I appreciate the extensive efforts the authors put in with many of the in vivo mouse studies presented here, but they reveal very little novel mechanistic insights.

We appreciate Reviewer 2’s honest assessment of our manuscript. While it is true that several of the results could be expected based on relatively recent publications, we believe that our work expands on several important concepts and mechanisms that contribute to an overall better understating of viral sensing pathways. While our study and Cheng et al.’s study share some results, our manuscript elucidates some novel details of the cGAS pathway, including the critical nature of the cGAS expression in the bone marrow compartment for optimal resistance to ECTV as well as the implication of IRF7 (and not IRF3) for optimal cGAMP anti-viral therapy.

Reviewer #3:

Specific issues:

1. In the 50:50 mixed B6-cGAS-/- bone marrow chimeras, the authors show that MHC-II^hi^ DCs, iMOs and NK cells were the main cell types impacted. Could they also comment on the distribution of other cells in the LN like T cells in these experiments.

The reconstitution of the mixed BM chimeras resulted in an approximately equal 50:50 distribution of CD45^+^ cells in the naïve contralateral LN (**Reviewer Figure 4**). The distribution of other immune cell types including B cells and T cells follow a similar trend as the MHC-II^hi^ DCs, iMOs and NK cells in *Cgas^-/-^* mice after infection, (approximately 50:50 CD45.1/*Cgas^-/-^* spread). Analysis of naïve LNs from *Cgas^-/-^* mice as well as the contralateral ndLNs of full and mixed BM chimeras revealed no inherent defects in the frequencies or total numbers of all of the immune cell subsets that we checked. Analysis of infected *Cgas^-/-^* mice at 1 dpi and 50:50 mixed BM chimeras revealed that cGAS expression is not required for recruitment or accumulation of immune cells into the dLN, however it renders cells more susceptible to infection and leads to smaller LNs and cellularity at 2 and 3 dpi. Therefore, the total number of different immune cell types, including B and T cells are decreased in the dLN later in infection, due to significantly smaller LNs.

***Reviewer Figure 4: Mixed CD45.1-Cgas^-/-^ BM chimera reconstitution profile.****.*

2. The authors comment that “cGAMP decreases viral replication in the skin ” however cGAS expression in non-hematopoietic cells has little effect on viral load in the spleen and liver measured at 7dpi (Figure 3). The viral gene expression in the dLN in the same figure is measured at 2dpi. What happens to viral loads in the spleen and liver at 2dpi dose? Is it possible that the role of cGAS in non-hematopoietic cells is dose dependent ? What is the ISG expression in skin in these chimeric mice?

There is no detectable virus in the spleen and liver at 2 days after ECTV inoculation into the footpad. 3 dpi is the earliest time point in which we have measured detectable virus in the spleen or liver. While it is true that cGAS expression in the non-hematopoietic compartment provides no apparent additive protective effect in terms of lowering viral replication in the dLN at 2 dpi (**Figure 3B**), virus titers at 7 dpi in the spleen and liver demonstrated a significant increase in viral burden in B6→*Cgas*^-/-^ BM chimeras compared to B6→B6 controls. Therefore, while cGAS expression in the BM compartment is required for optimal protection, cGAS expression in the non-hematopoietic compartment may play a small protective role. Furthermore, cGAMP administration into the skin followed by ECTV infection induced a massive upregulation of IFN-I/ISGs in the skin, which, in addition to decreasing local infection, likely curbs viral dissemination.

3. The authors mention that the decreased IFN-I expression and increased infection rate of MHC-II^hi^ DCs and iMOs in the dLN of Cgas^-/-^ mice could increase viral-induced cell death without affecting frequencies of MHC-II^hi^ DCs and iMOs. Do the authors have a way to quantify this ?

Unfortunately, we do not exactly have a direct way to quantify this, we came to this postulation from the entirety of our experimental data. From 1, 2 or 3 dpi, we have always observed no significant changes in MHC-II^hi^ DC, iMO, or NK cell frequencies (**Figure 4B, K,** and **S**) but significantly lower total numbers at 2 and 3 dpi, but not 1 dpi (**Figure 4C, L,** and **T**). This is due to smaller total cell numbers in *Cgas^-/-^* dLNs at 2 and 3 dpi, which is correlated with significantly higher infection rates of all measured immune cell types (**Figure 4F, O,** and **U**) and higher levels of cell death in the dLN (**Figure 4V**). Thus, our data suggests that cGAS deficiency is not required for immune cell accumulation in the dLN but renders cells more susceptible to infection, most likely due to inability to properly sense viral infection and signal IFN-I production, consequently leading to accelerated viral infection and cell death.

4. Figure 5 points out that administration of cGAMP 4 hrs prior to ECTV infection restores viral resistance however the gene expression data and cellular numbers in LN are not convincing.
“However, there was a drastic reduction in Ifna4 (Figure 5C) Ifna-non4 (Figure 5D) and Ifnb1 (Figure 5E), as well as a significant reduction in total numbers of MHC-II^hi^ DCs (Figure 5F) and iMOs (Figure 5G).” Please indicate what is the comparison here. Have the authors looked into cell frequencies in LN after cGAMP + ECTV? What about viral load in spleen and liver in ECTV+cGAMP conditions? What cells are infiltrating the skin after cGAMP administration?

The flow of Figure 5 followed our initial train of thought as we obtained our results. After we observed 100% protection with cGAMP administration and drastically reduced viral replication in the dLN, we hypothesized that that was probably due to significantly increased MHC-II^hi^ DC recruitment from the skin to the dLN due to cGAMP priming and concomitant increase in iMO and IFN-I response. While we saw a significant increase in MHC-II^hi^ DC migration to the dLN after cGAMP administration (**Figure 5F**), we were initially surprised to see decreased iMO numbers (Figure 5G) and IFN-I expression (**Figure 5C-E**) in the dLN. However, cGAMP administration induces a large increase in IFN-I and ISGs in the skin (**Figure 5I-O**), therefore increasing resistance to viral propagation and therefore significantly dampening viral dissemination and spread to the dLN. This significant reduction in virus in the dLN most likely accounts for the significantly reduced numbers of iMO. We observed a significant increase in CD64^+^ cells in the skin (mostly monocytes and neutrophils) 2 days after cGAMP administration (**Reviewer Figure 5C**). Moreover, there was a significant increase in NK cells in the skin of *Cgas^-/-^* mice compared to B6 at 2 days. We have edited the text in the Results to provide more clarity.

***Reviewer Figure 5: Immune cell recruitment to the skin after infection and cGAMP administration.*** *Data are displayed as mean ± SEM with 6-9 mice per group combined from two similar, independent experiments. For all, **p<0.01*

5. cGAMP administration to IRF7 KOs does significantly change a set of genes in ECTV+cGAMP condition as compared to ECTV+cGAMP in B6 such as Ifna4 (skin Fig 6C) , Ifna-non4 in skin and dLN (Figure 6 E and H). A set of genes Ifit3, Isg15 and Mx1 were only tested in skin and not in LN. It’s not clear from the data presented if all the effects are IRF7 dependent? Why do a set of genes change and others don’t ? This is observed in other figures too, Figure 5J in B6 mice ECTV compared to ECTV+cGGAMP ifna4 is significantly upregulated but ifna-non4, Ifnb1 are unchanged. cGAMP administration in B6 mice would likely enhance all ISGs.

The expression of ISGs in the dLN of *Irf7^-/-^* mice mirror that of the skin, in that cGAMP administration was unable to induce ISG induction in the dLN due to IRF7 deficiency. We have edited the text in the Results to acknowledge this. Our previous study demonstrated that sequential pathways downstream of STING mediate differential IFN-I expression as NF-kB signals for IFN-β and IRF7 signals for IFN-α (specifically IFN-α5) specifically in iMO in the dLN, which are the main producers of IFN-I after ECTV infection (Xu et al. 2015). The skin and dLN environments are very different in terms of their cellular and structural composition, therefore that could very well affect the specificity and magnitude of the IFN-I response. Furthermore an additional caveat is that we are measuring IFN-I expression at 2 dpi so the level of virus in the skin and dLN are going to be different as well, which could also contribute to the differences in IFN-I expression.

6. What is the status of MHC-IIhi DCs and iMOs (numbers or frequencies) in dLN in IRF7 or IFNAR deficient mice treated with cGAMP?

ECTV-infected *Irf7^-/-^* mice treated with cGAMP have reduced numbers of MHC-II^hi^ DCs compared to B6 controls (**Reviewer Figure 6A**). Infected B6 mice have significantly higher accumulation of iMO in the dLN compared to *Irf7^-/-^* mice (**Reviewer Figure 6B**). While B6+cGAMP and *Irf7^-/-^* +cGAMP have comparable numbers of iMO in the dLN at 2 dpi (**Reviewer Figure 6B**), there are significantly higher levels of viral replication in *Irf7^-/-^* mice+cGAMP in the dLN (**Figure 6B**), therefore the lower numbers of iMO in B6+cGAMP may be attributed to less viral burden at the same measured time points. Moreover, *Irf7^-/-^* mice treated with cGAMP have a small but significant increase in iMO (**Reviewer Figure 6B**), which could contribute to the slight but not statistically significant increase in survival rates. *Ifnar1^-/-^* mice treated with cGAMP have no significant differences in MHC-II^hi^ DC numbers in the dLN at 2 dpi (**Reviewer Figure 6C**). However, infected *Ifnar1^-/-^* mice have significantly lower total numbers of iMO and while there is no difference in iMO numbers in the cGAMP-treated groups, a similar discrepancy in viral replication levels exist between B6 and *Ifnar1^-/-^* mice (**Reviewer Figure 6D**).

***Reviewer Figure 6: MHC-II^hi^ DC and iMO accumulation in the dLN of Irf7^-/-^ and Ifnar1^-/-^ mice.*** *Data are displayed as mean ± SEM with 8-10 mice per group combined from two similar, independent experiments. For all, *p<0.05, **p<0.01, ***p<0.001.*

7. In Figure 7 C and D why ifna-4 and Ifna-non4 are increased in the skin but not in dLN of IFNAR1 deficient mice , comparing ECTV +cGAMP in B6 to ECTV+ cGAMP in IFNAR KO? Are there different cell types infiltrating these two tissues after cGAMP administration?

We believe that Reviewer #3 accidently misinterpreted the results as both infection alone and infection+cGAMP in *Ifnar1^-/-^* mice induces a large increase in IFN-I expression in both the skin (**Figure 7C-E**) and dLN (**Figure 7F-H**) at 2 dpi, but there is a lack of ISG expression in the skin (**Figure 7I-L**), even after cGAMP administration. This confirms that IFN-I signaling through IFNAR1 is required for the upregulation of ISGs and the anti-viral state, and that their absence lead to increased dissemination and viral replication in the dLN.

Part III – Minor Issues: Editorial and Data Presentation Modifications
Reviewer #1: Headline: As the manuscript is only about one single type of virus – then I think “an acute viral disease” should be replaced by the name of the actual disease/infection.

We have amended “acute viral disease” to “Ectromelia virus infection” in the title.

The two separated sections that both refer to figure 1 should be merged.

We have merged the two sections referencing Figure 1 and amended the title for Figure 1 in the Results.

Fig 4G: Could the authors clarify what this panel measures. Is it uninfected mice that have such a high level of evm003 expression?

Figure 4G is measuring relative mRNA expression levels of viral gene *Evm003* from sorted “infected” (ECTV-dsRED^+^) MHC-II^hi^ DCs and “uninfected” (ECTV-dsRED^-^) MHC-II^hi^ DCs from the dLNs of B6 and *Cgas^-/-^* mice 2 dpi. The relative fold change is compared to pooled contralateral naïve LNs. Reviewer 1 does bring up an astute point in that there are relatively high *Evm003* levels in “uninfected” MHC-II^hi^ DCs and we are attributing that to a pool of DCs that may be in the earlier stages of infection where they do not express detectable levels of ECTV-dsRED but still harbor some virus. This seems plausible as cGAS deficiency renders most immune cell types more susceptible to higher levels of viral infection.

Figure 5:
This figure is not really informative beside the point that cGAMP can induce IFN-I, which we already know can protect from infection. Admin of other IFN-I inducing agents, or IFN-I itself, is likely to have same effect. Therefore, these experiments neither support nor stand against the hypothesis that Cgas is important for the resistance.

While it is true that the results in Figure 5 demonstrating exogenous cGAMP administration induces IFN-I and ISG expression and confers protective efficacy are not surprising, we still believe it is an integral part of the story through the demonstration of **1)** Complete rescue of susceptible *Cgas^-/-^* mice from lethal challenge, **2)** Expands on the dynamics of viral spread and curbing of dissemination by cGAMP-mediated IFN-I protection and **3)** Sets up the rest of the manuscript in which we identify the specific transcription factors and receptors that are critical for cGAMP-mediated therapy.

Figure 6: From Figure 6M it is clear that the effect of cGAMP is only partly dependent on Irf7. This should be reflected in the figure caption.

Based on the reviewer’s recommendation, we have added “mostly” to the subtitle and figure caption.

Reviewer #2: Some minor comments:
1. cGAS knockout mice has reduced ISG expression at baseline. Some of the ISG measurements (e.g. Figure 2) need to show before and after infection values. The 2-fold decrease in most ISGs after infection may be similar to that of before infection, if so, that will complicate the interpretation of ISGs.

We have actually taken this caveat into consideration and all of the relative fold changes are compared to each group’s corresponding naïve dLN. We have emphasized this more in the text to avoid any confusion or ambiguity.

2. Poxvirus antagonize cGAS pathway through poxins that cleaves cGAMP (PMID: 30728498). Does ECTV encode a poxin to antagonize cGAMP?

The VACV gene B2R described in PMID: 30728498 is absent in ECTV (PMID: 14675635). Yet, whether other cGAMP cleaving enzymes exist in ECTV is, to our knowledge, unknown.

3. The discussion is too long. Much of the content is comparing the Cheng et al study rather than highlighting the implication of some of the findings.

We have edited the discussion to be more succinct, with more of an emphasis on the importance and implications from our results.

Reviewer #3: Throughout the manuscript its difficult to follow which conditions are being compared to draw clear conclusions. Additional information in legend or text would help the reader. Please state what conditions are controls and what conditions are being tested.

We have made edits to improve the flow and clarity of the manuscript.
